# COVID-19 knowledge, attitudes and practices in a conflict affected area of the South West Region of Cameroon

**DOI:** 10.11604/pamj.supp.2020.35.2.22963

**Published:** 2020-05-13

**Authors:** Tendongfor Nicholas, Forlemu Vanessa Mandaah, Seraphine Nkie Esemu, Amana Bokagne Theresa Vanessa, Kouam Talla Destin Gilchrist, Lambou Fopa Vanessa, Nsagha Dickson Shey

**Affiliations:** 1 Department of Public Health and Hygiene, University of Buea, P.O. Box 63, Buea, Cameroon; 2 Department of Microbiology and Parasitology University of Buea, P.O. Box 63, Buea, Cameroon; 3 Laboratory for Emerging Infectious Diseases, University of Buea, P.O. Box 63, Buea, Cameroon; 4 Department of Biochemistry of Molecular Biology, University of Buea P.O. Box 63, Buea, Cameroon

**Keywords:** COVID-19 pandemic, conflict areas, knowledge gap, attitude, practices, Buea Health District

## Abstract

**Introduction:**

since December 2019, the world is experiencing, the COVID-19 pandemic caused by the Virus SARS-CoV-2. It is feared that the pandemic maybe more devastating in conflict affected areas in the world. This study assessed the knowledge, attitudes and practices with regard to the COVID-19 in Buea municipality, Cameroon.

**Methods:**

this was a cross-sectional study carried out in communities selected from 5 health areas of the Buea Health District. A questionnaire was administered to collect data on the knowledge, attitudes and practices on COVID-19. The knowledge was measured using a 26-points Liker scale on awareness, mode of transmission, clinical manifestation, site of the infection and prevention. The data was analyzed in SPSS version 25.

**Results:**

of the 545 particpants who consented, 21.9% had a correct knowledge of COVID-19, 43.8% had intermediate knowledge, 34.4% had poor knowledge and 11.93% had no knowledge. Majority of the participants (73.3%) knew they were at risk of contacting the infection. They were aware that cleaning and disinfecting the environment (78.8%), reducing contact with animals (56.3%) could help prevent the infection. Even though participants knew about the face mask (93.5%) and hand sanitizers (88.8%), only 21.7% and 32.9% had purchased them respectively. Few participants reported that they will go to a man of God (6.0%), native doctor (3.5%) and auto-medication (25.8%) if infected.

**Conclusion:**

There is still a knowledge gap in the Buea population with respect to COVID-19. The population is aware of the disease and preventive measures, but few have taken steps to procure essential tools for the prevention. There is need to intensify sensitization to fill the knowledge gap the population has with regards to COVID-19.

## Introduction

In December 2019, a new coronavirus emerged in China causing an acute respiratory disease known as coronavirus disease 2019 (COVID-19) [1]. The virus was identified to be a betacoronavirus related to severe acute respiratory syndrome coronavirus (SARS-CoV) and thus was named Severe acute respiratory syndrome corona virus 2 (SARS-CoV-2) [2]. In two decades, this virus is the third known coronavirus to cross the species barrier and cause severe respiratory infections in humans after SARS-CoV in 2003 and Middle East respiratory syndrome coronavirus virus (MERS-CoV) in 2012 [3,4]. SARS-CoV-2 was first detected in Wuhan City, Hubei Province, China and has spread to almost all countries in the world. On the 30th of January 2020, the International Health Regulations Emergency Committee of the World Health Organization (WHO) declared the outbreak a “public health emergency of international concern” (PHEIC) [5]. The World Health Organization declared the disease a pandemic on the 11th March 2020 [6] and most countries worldwide have registered cases of COVID-19 including Cameroon. Recent statistics show more than 2.3 million confirmed cases worldwide with more than 13,000 confirmed cases from Africa [7]. African countries as a whole and Cameroon in particular are characterized by poor health systems with low expertise to combat such a virus. In Cameroon the first case was reported on the 6th of March 2020 [8] and a month after, more than 1000 people have been infected with SARS-CoV-2 [9].

The rapid spread of this virus and its pandemic nature requires therefore a global awareness of the disease and population preparation to face an outbreak. The SARS-CoV-2 has been very devastating in western countries which are characterized by a very well-structured health system. It is feared that its spread to Africa may be more devastating because of the poor health systems. The Cameroon culture is generally characterized by tight family relationships with people making several contacts per day. This pandemic may further be exacerbated in the anglophone regions of Cameroon as a result of the ongoing conflict that has led to a breakdown of the health system in these regions. The best tool that African countries may use to avoid a potential humanitarian disaster caused by the SARS-CoV-2 virus is to strictly implement preventive measures which have proven their efficacy in other countries like China where the disease started. However, preventive measures can be well implemented only if the population has good knowledge about the disease and its public health importance. With several cases already confirmed in Cameroon, the government in addition to the WHO guidelines [10] put in place a series of measures to prevent the spread of the infection. Among these measures are: closing of schools, closing of international borders, wearing of face masks, regular hand washing, use of hand sanitizers, creation of an emergency number and measures to prevent social contacts. In this study, we assessed the level of the knowledge and awareness of the Buea population with respect to the COVID-19 pandemic as well as their attitude and practices towards the pandemic.

## Methods

**Study site:** the study was carried out in Buea, the administrative head quarter of the South West region of Cameroon, one of the regions affected by the Anglophone crisis since 2016. Buea is a town located on the eastern slopes of Mount Cameroon and lies between latitudes 40 12´N and longitudes 90 12´ E with a total surface area of 870 km2. The total population estimated by the health population denominators (2013) revealed 300,000 inhabitants [11]. The population is mainly made up of students and civil servants who are highly concentrated in Molyko. Many internally displaced people from neighbouring villages affected by the Anglophone crisis since 2016 have found refuge in Buea municipality. Buea is one of the fastest growing towns in Cameroon today with a mix cosmopolitan setting. The epidemiological data was collected in communities selected in 5 health areas (Buea Road, Molyko, Muea, Bokwango and Tole) out of the eight health areas in the Buea Health District. The choice of these areas for the sample collection was motivated by the accessibility, security and population density.

**Study design:** this was a cross-sectional study carried out in five health areas of the Buea health district between march 10th-18th 2020. A questionnaire was administered to collect data on the knowledge, attitude and practice of the population on COVID-19 pandemic.

**Study population:** the study population was made up of males and females, age 18 years and above residing in the Buea Municipality. Participants were recruited from households and hot spots (population gathering points) in different communities selected from five health areas. All those who consented by signing the consent form were interviewed. A sample size of 384 was determined using the Lorenz formula with an expected proportion of the population having accurate knowledge of the virus adopted from a study conducted on a related viral infection (SARS) carried out in 2004 [12].

**Sampling collection tools:** the data collection tool was a closed-ended questionnaire adapted from the WHO guidelines on clinical manifestation and Prevention of the COVID-19 [13]. The questionnaire was pretested in Bova (a health area in the Buea municipality which was not included in the study). After the pre-testing, the questionnaire was validated by the research team. This consisted in restructuring questions that were not understood by the participants or poorly structured. The questionnaire consisted of socio-demographic data, participant´s awareness of COVID-19, knowledge on mode of transmission, signs and symptoms and the knowledge of preventive measures.

**Sampling technique:** this study was a two-stage clustered (health area and community based) cross-sectional questionnaire-based survey. Five health areas were purposefully selected based on accessibility, security and population density. Data was collected in communities selected from the five health areas. In each health area, two to five communities were randomly selected. At the level of the community, data was collected from households and hot spots (meeting points, motor parks, restaurants and workshops). In each household visited at most two persons were interviewed. Care was taken to include in the sample individuals of different age groups, gender, education and profession. Individuals who had no knowledge of the COVID-19 outbreak were excluded from the study. The questionnaire was administered to participants in English. Participants who were literate were allowed to fill the questionnaire themselves whereas those who could not read or write, the questionnaire were administered in “Pidgin English”, a local language commonly spoken and understood in the area.

**Data analysis:** the data collected were entered into a template created in Epi Info version 7.2 and analyzed in the Statistical Package for the Social Sciences (SPSS) version 25 (SPSS, Inc., Chicago, IL, USA). The data were analyzed and summarized using descriptive statistics and the results were displayed in charts and tables. A p-value < 0.05 was considered statistically significant. The assessment of the awareness of the COVID-19 was done using the following: participant knowledge of the existence of the disease, name of the virus, reservoir, the country of origin, other countries affected, site of infection, presence of the disease in Cameroon and first town where the disease was reported in Cameroon. Participants´ knowledge on transmission was measured using 4-point checklist comprising: direct contact, contact with infected objects, respiratory droplets (particles sneezed or coughed into the air), touching of infected surfaces. Participants´ knowledge on method of prevention was measured using a 10-point checklist developed from the WHO guidelines [10] which comprised of: avoiding contact with infected people, touching the eyes and nose, staying home, observe social distancing, covering the mouth when coughing, avoiding hand shaking, sneezing in the fold of the elbow, wearing of face mask, regular washing of hands with soap, disinfecting touched objects and surfaces. For the knowledge of clinical manifestation, participants were assessed using a 5-point checklist including: fever, running nose, cough, shortness of breath and sore throat. Each correct response above was scored 1 point, otherwise 0. In total, the maximum obtainable score was 26 points. Those who scored 1 to 8 point(s) were considered having low knowledge, 9-16 were considered having intermediate knowledge and above 16 points were considered having correct knowledge. The association between the level of knowledge and socio-demographic characteristics of the participants was compared using a chi-squared test.

**Ethical considerations:** ethical clearance (2020/1203-04/UB/SG/IRB/FHS) was obtained from the Institutional Review Board of the Faculty of Health Sciences, University of Buea. An administrative clearance was gotten from the Regional Delegation of Public Health, South West Region. The various participants gave their consent before being enrolled into the study. For participants less than 21 years, an assent was obtained from the participant in addition to the consent obtained from the parents or guardians.

## Results

Socio-demographic characteristics of the study participants: a total of 480 participants were interviewed, among which 211 (44.0 %) were females and 269 (56%) were males. The age of participants ranged from 18 to 70 years with mean (SD) age 30.03 (11.2) years. Participants were recruited from Bokwango (18.8%), Buea Road (25.0%), Molyko (22.9%), Muea (20.8%) and Tole (12.5%). Majority of the participants were single (59.4 %), had attended tertiary education (55.6%) and were Christians (92.7%). Of the study participants, 236 (49.2%) were employed and 198 (41.3%) were students. In terms of household size, 198 (41.3%) came from household with 4 to 6 inhabitants and 132 (27.5%) from household with 2 to 3 inhabitants (Table 1).

**Table 1:** Socio-demographic characteristics of the study participants

Variable	Levels	Frequency	Percent
**Health area**	Bokwango	90	18.8
	Buea Road	120	25.0
	Molyko	110	22.9
	Muea	100	20.8
	Tole	60	12.5
**Gender**	Female	211	44.0
	Male	269	56.0
	**Mean**	**30.03**	
**Age group (years)**	< 20	49	10.2
	20 - 29	237	49.4
	30 - 39	114	23.8
	40 - 49	47	9.8
	50 +	33	6.9
**Marital Status**	Co-habiting	22	4.6
	Divorced	6	1.3
	Married	167	34.8
	Single	285	59.4
**Education**	No formal education	25	5.2
	Primary	22	4.6
	Secondary	166	34.6
	Tertiary	267	55.6
**Religion**	Christian	445	92.7
	Muslim	21	4.4
	Pagan	14	2.9
**Profession**	Employed	236	49.2
	Student	198	41.3
	Unemployed	46	9.6
**Household size**	1	48	10.0
	2-3	132	27.5
	4-6	198	41.3
	> 6	102	21.3

Level of awareness of COVID-19: a total of 545 participants consented of which 65(11.92%) did not know about the COVID-19 and were not included in the study. Of the 480 participants interviewed 443 (93.2%) knew the name and the country where the disease was first reported, 370 (77.1) reported a virus as causative agent and 291 (60.6 %) said that the disease was from animal origin. A majority of the participants got informed about the disease from the television 308 (64.2%) and social media 184 (38.8%). Only 105 (21.9%) participants did not know any other country affected by the disease. About the participant awareness of the disease in Cameroon, 305 (63.5%) acknowledged the disease was already in Cameroon out of whom 274 (57.1%) cited Yaoundé as the town of origin of the infection in Cameroon (Table 2). About the site of infection, 394 (82.1%) participants reported the respiratory tract as the site of the infection, whereas some participants reported other sites including the brain (2.5%), skin (12.7%), and Gastrointestinal tract (6.7%). Most of the clinical manifestations reported by participants were cough (76.5%), fever (53.1%), running nose (36.5%), sore throat (34.8%), shortness of breath (32.7). Others clinical manifestations such back pain (4.8%) and stomach pains (9.0%) were also reported by participants (Table 3). For the mode of transmission of the disease, 358(74.6 %) of the participants reported that the transmission of the disease was by direct contact, 217(45.2%) reported that it was via contact with infected objects while 249(51.9%) reported that transmission was through droplets sneezed in the air. Few participants reported transmission by sexual contact, eating of contaminated food and by blood (Table 3). All participants knew at least one preventive measure of the disease, but the number of preventive measures sited varied from one participant to the other. Most of them reported avoiding close contact with patients, 394(82.1%), whereas the least mentioned mode of prevention was coughing and sneezing into the fold of the elbow, 99(20.6%). Few participants reported transmission by food, blood and sexual contact (Table 3).

**Table 2:** Level of awareness of the study participants

Participant awareness of COVID-19		Frequency	Percent
Name of the disease	HIV	4	0.8
	COVID-19	443	92.3
	EBOLA	28	5.8
	Influenza virus	4	0.8
Reservoir	Human	189	39.4
	Animal	291	60.6
Source of information	Television	308	64.2
	Newspapers	41	8.5
	Internet	184	38.3
	Friends	91	19.0
	Family	45	9.4
Country of origin	China	443	92.3
	Others	36	7.5
Knowledge of other country affected by COVID-19	No	105	21.9
	Yes	375	78.1
Causative agents	Bacteria	72	15.0
	Fungi	36	7.5
	Viruses	370	**77.1**
	Parasites	8	1.7
Any case registered in Cameroon so far?	Yes	305	63.5
	No	175	36.5
Knowledge of town of first reported case in Cameroon	Yes	274	57.1
	No	206	42.9

**Table 3:** Participant's knowledge of mode of infection, clinical Manifestations and prevention of the disease

Variables	Levels	Frequency	Percent
**Site of infection**	Respiratory tract	394	82.1
	GIT tract	32	6.7
	Brain	12	2.5
	Droplets	12	2.5
	Skin	61	12.7
**Symptoms of the infection**	Fever	255	53.1
	Cough	367	76.5
	Sore throat	167	34.8
	Running nostrils	175	36.5
	Shortness of breath	157	32.7
	Backpain	23	4.8
	Stomach pain	43	9.0
**Mode of transmission**	Contact with infected persons	358	74.6
	Contact with infected objects	217	45.2
	Sexual transmission	54	11.3
	Respiratory tract	110	22.9
	Eating of contaminated food	108	22.5
	Particles sneezed or coughed in the air	249	51.9
	Blood	59	12.3
**Mode of prevention**	Avoid close contact with patients	394	82.1
	Avoid touching eyes, nose and mouth	209	43.5
	Stay home	192	40.0
	Cover cough	206	42.9
	Disinfect objects	242	50.4
	Regular hand washing	284	59.2
	Cough and sneeze in the elbow	99	20.6
	Greet people without shaking hands	221	46.0
	Social distancing	152	31.7
	Wearing of face mask	268	55.8

**Figure 1:**
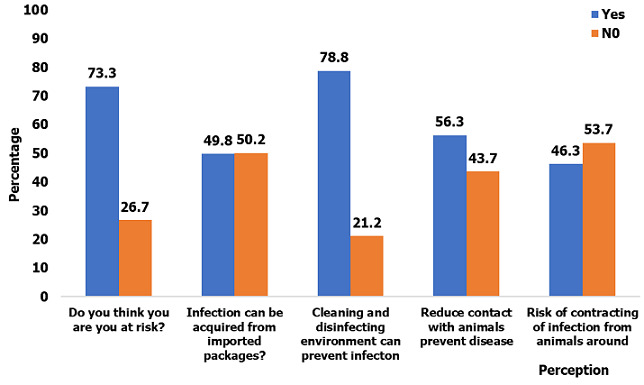
Perception of participants towards COVID-19

Participant´s attitude and perception towards COVID-19: participants were asked a question on what to do if they were infected with COVID-19. The following responses were obtained: calling the emergency number (75.6%), wearing of face masks (50.2%), avoiding contact with others (49.6%), going for prayers (6.0%), going to a native doctor (3.5%) and auto-medication (25.8%). On their perception of the disease, 73.3% acknowledged that they were at risk of contracting the infection, 78.8% said that cleaning and disinfecting the environment could help prevent the infection. Whereas 46.3% recognized they were at risk of contracting the disease from animals and 56.3% acknowledged that reduced contact with animals can help prevent the infection. Some participants (49.8%) reported that individuals could be infected via packages shipped from infected countries (Figure 1).

**Figure 2:**
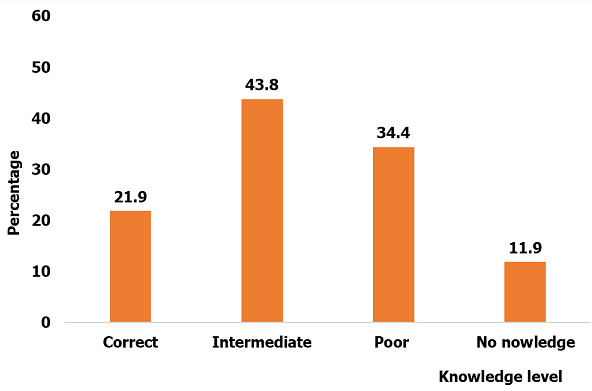
Level of knowledge of the study participants on COVID-19

Participant level of knowledge on COVID-19: the participants level of knowledge was categorized into correct, intermediate and poor knowledge using a liker scale (Figure 2). Of all the study participants, 21.9% had a good knowledge of the disease (scored 16 points and bove), 43.8% of them had an intermediate knowledge (scored 9-15 points) and 34.4% had poor knowledge of the disease (scored less than 9 points). No association was observed between the level of knowledge and socio-demographic charateristics (Table 4).

**Table 4:** Relationship between the level of knowledge and the socio-demographic characteristics

Variable	Level	Correct (%)	Intermediate (%)	Poor (%)	Chi-square	P-value
**Health area**	Bokwango	15 (3.1)	41 (8.5)	34 (7.1)	12.89	0.116
	Buea Road	24 (5.0)	53 (11.0)	43 (9.0)		
	Molyko	22 (4.6)	48 (10.0)	40 (8.3)		
	Muea	30 (6.3)	48 (10.0)	22 (4.6)		
	Tole	14 (2.9)	20 (4.2)	26 (5.4)		
**Age group (Years)**	< 20	15 (3.1)	15 (3.1)	19 (4.0)	13.51	0.095
	20 - 29	52 (10.8)	94 (19.6)	91 (19.0)		
	30 - 39	21 (4.4)	61 (12.7)	32 (6.7)		
	40 - 49	10 (2.1)	26 (5.4)	11 (2.3)		
	50 +	7 (1.5)	14 (2.9)	12 (2.5)		
**Gender**	Female	52 (10.8)	89 (18.5)	70 (14.6)	1.69	0.430
	Male	53 (11.0)	121 (25.2)	95 (19.8)		
**Marital status**	Co-habiting	3 (0.6)	12 (2.5)	7 (1.5)	4.7	0.581
	Divorced	1 (0.2)	3 (0.6)	2 (0.4)		
	Married	35 (7.3)	81 (16.9)	51 (10.6)		
	Single	66 (13.8)	114 (23.8)	105 (21.9)		
**Education level**	None	5 (1.0)	9 (1.9)	11 (2.3)	3.12	0.793
	Primary	6 (1.3)	11 (2.3)	5 (1.0)		
	Secondary	34 (7.1)	77 (16.0)	55 (11.5)		
	Tertiary	60 (12.5)	113 (23.5)	94 (19.6)		
**Profession**	Civil service	22 (4.)	42 (8.8)	27 (5.6)	14.85	0.062
	Farmers	7 (1.5)	23 (4.8)	7 (1.5)		
	Private sector	19 (4)	56 (11.7)	37 (7.7)		
	Student	48 (10.0)	76 (15.8)	74 (15.4)		
	Unemployed	9 (1.9)	13 (2.7)	20 (4.2)		
**Religion**	Christian	103 (21.5)	187 (39.0)	155 (32.3)	9.56	0.48
	Muslim	2 (0.4)	13 (2.7)	6 (1.3)		
	Pagan	0 (0.0)	10 (2.1)	4 (0.8)		
**Household size**	1	8 (1.7)	22 (4.6)	18 (3.8)		
	2 - 3	26 (5.4)	49 (10.2)	57 (11.9)	8.08	0.232
	4 - 6	47 (9.8)	93 (19.4)	58 (12.1)		
	> 6	24 (5.0)	46 (9.6)	32 (6.7)		

Participants knowledge on measures taken by the government to prevent spread of COVID-19: it was observed that participants were less aware of the measures taken by the government to prevent the spread of the virus. The most sited measures included those that affected them directly like closing of schools (34.2%) and avoiding social gathering (31.7%). Few participants cited government measures such as closing of borders (25.4%), wearing of face mask (2.7%), calling of emergency number (2.1%), hygiene and sanitation, quarantine centres (1.9%), screening of patients (0.6%), and 0.6% had no idea of any measures taken (Table 5).

**Table 5:** Knowledge of participants on the various measures taken by the Government

Variable	Level	Frequency	Percent
**Measures taken by the government?**	No action	3	0.6
	Closing of schools	164	34.2
	Wearing of Face masks	13	2.7
	Closing of borders	122	25.4
	Avoid social gathering	152	31.7
	Emergency number	10	2.1
	Hygiene and sanitation	9	1.9
	Quarantine centre	9	1.9
	Screening of patients	3	0.6

## Discussion

The knowledge, attitude and practices of a particular population with respect to an infectious illness can be influenced by various factors namely, the gravity of the illness, severity of its spread and the fatality rate [6]. Ever since the announcement of COVID-19 as a pandemic by the WHO [14], there has been growing knowledge and awareness towards the disease as it spreads. The first case of COVID-19 was reported in Cameroon on March 6th 2020 [8] and since then the number of cases has risen steadily to above 1000 at the time this manuscript is written.

Participant knowledge of COVID-19: it was observed that the level of knowledge of the Buea population did not vary from one health area to the other and with socio-demographic characteristics. Only 21.9% of the study population had a correct knowledge of the infection with a small fraction of the population approached knowing nothing about the disease. Despite the fact that the pandemic has been ongoing for three months prior to this study, there was still a great knowledge gap in the population of the Buea municipality. This knowledge gap could be due to the fact that by the time this study was conducted, only few cases were reported in Cameroon and none in the South West region or in the Buea municipality where we carried out the study. This knowledge gap could also be due to the fact that the Buea population is living in an area affected by the Anglophone crisis and many people may still be adjusting to accommodate the COVID-19. These findings differ from those of a similar study conducted on a related viral infection (SARS-CoV) in which respondents demonstrated good knowledge of the infection [12]. The knowledge gap is an indication that more measures are needed to be put in place to ensure proper sensitization of the population with emphasis in conflict areas where people are already traumatized by the conflict. It is likely that the knowledge gap will decrease with the progression of the disease. As of date, the government has reinforced several measures to prevent the spread of the infection and we envisage that this knowledge gap will reduce as the disease spreads in Cameroon.

Attitudes and practices: even though few people had correct knowledge of the COVID-19, there was generally a good attitude of the population towards the disease like calling the emergency number and reporting to the hospital if sick or having a suspected case of COVID-19 at home. Few deviations were observed in some participants like resorting to auto-medication, prayers and traditional healers in the case of an infection. A good proportion of the participants had a good attitude towards implementing the WHO guidelines. These findings were similar to those reported by Hamed Alzoubi1 [6], in a Knowledge, attitude and practice (KAP) study carried out on COVID-19 on medical and non-medical university students of Mu´tah University in Jordan. Most of the participants knew they were at risk of contracting the disease and were aware that reduced contact with animals and keeping their environments clean could reduce their risk of contracting the disease, but failed to procure essential tools such as face masks and hand sanitizer for their protection. This could be due to the fact that by the time this data was collected, the government had not yet taken radical measures like compulsory wearing of face mask and provision of hand washing facilities in public places.

Participants knowledge on Government response to prevent spread of COVID-19: measures taken by the government to prevent the COVID-19 were poorly known by the participants at the time of data collection. They knew only measures that affected them directly such as closing of schools and reduction of social gatherings. Even though participants were quite aware of preventive measures such as wearing of face mask, hygiene and sanitation and other measures, they failed to recognize these as part of the measures recommended by the government. This portrayed some level of disconnection between the population and the government measures, justifying the low implementation of these measures at the time this data was collected. In a related study carried out in Saudi Arabia [15] there was a high population compliance to government measures. Reinforcement of government measures, community-based sensitization and health education programs will go a long way to prepare the community to overcome the pandemic. Providing this education to the population will help fill some knowledge gaps and correct some misperception regarding the attitude of the population towards the disease. With the recent statistics showing more than 13000 cases in Africa and more than 1000 cases in Cameroon [9], it is feared that the SARS-CoV-2 spread may be more devastating in the days ahead especially in countries plagued with conflicts like Cameroon characterized by a seriously affected health system. The Cameroon culture, mainly characterized by tight family closeness with people making several contacts per day, may be another exacerbating factor to the spread of the infection. Thus, strict observation of the preventive measures maybe the best option for Cameroonians to reduce the impact or to control the COVID-19 pandemic in the entire nation especially in conflict affected areas.

## Conclusion

This study outlined some knowledge gaps in the population of Buea with respect to the COVID-19 pandemic. Even though majority of the population was aware of the disease and its risks, few had acquired essential tools such as face masks and sanitizers for personal protection. There is need to reinforce preventive measures and intensify sensitization campaigns to fill the knowledge gap of the population with regards to COVID-19. Without confining the country, the government of Cameroon is implementing several measures to curve down the pandemic and there is a need to evaluate the impact of these measures on the knowledge and practices of the population with the progression of the pandemic in Cameroon.

**Limitation of the study:** one of the limitations of the study is that a qualitative arm was not included.

**What is known about this topic**

- The first cases of Covid-19 was declared in Cameroon on the 6th of March 2020;
- The government of Cameroon has put in place 20 measures to prevent the spread of the disease in Cameroon;
- More than 1000 cases have been diagnosed in Cameroon one month after the first case and so far more than 40 deaths recorded.

**What this study adds**

- More than 1000 cases have been diagnosed in Cameroon one month after the first case and so far more than 40 deaths recorded.
- The population had a good attitude towards the disease, but only few had acquired basic protective tools such as face masks and hand sanitizers;
- Poor knowledge of the government measures to prevent the spread of the SARS-CoV-2 pandemic in Cameroon.

## Competing interests

The author declares no competing interests.

## References

[1] Zhou Peng, Yang Xing-Lou, Wang Xian-Guang, Hu Ben, Zhang Lei, Zhang Wei (2020). A pneumonia outbreak associated with a new coronavirus of probable bat origin. Nature.

[2] Wu Yuntao, Ho Wenzhe, Huang Yaowei, Jin Dong-Yan, Li Shiyue, Liu Shan-Lu (2020). SARS-CoV-2 is an appropriate name for the new coronavirus. The Lancet.

[3] Perlman Stanley (2020). Another decade, another Coronavirus. N Engl J Med.

[4] Huang Chaolin, Wang Yeming, Li Xingwang, Ren Lili, Zhao Jianping, Hu Yi (2020). Clinical features of patients infected with 2019 novel coronavirus in Wuhan, China. Lancet.

[5] World Heath Organization Novel Coronavirus(2019-nCoV) Situational Report-11.

[6] Alzoubi1 Hamed, Alnawaiseh Nedal, Al-Mnayyis Asma´a, Abu- Lubad Mohammad, Aqel Amin, Al-Shagahin Hani (2020). COVID-19-Knowledge, Attitude and Practice among Medical and Non-Medical University Students in Jordan. J Pure Appl Microbiol.

[7] COVID-19 Visualizer.

[8] World Heath Organization Coronavirus disease 2019 (COVID-19) Situational Report-46.

[9] World Heath Organization Coronavirus disease 2019 (COVID-19) Situation Report-89.

[10] WHO (2020). Coronavirus disease (COVID-19) advice for the public.

[11] Helders and Stefan Buéa. World Gazetteer.

[12] Bener Abdulbari, Al-Khal Abdullatif (2004). Knowledge, attitude and practice towards SARS. The Journal of the Royal Society for the Promotion of Health.

[13] CDC Coronavirus Disease 2019. Information for Healthcare Professionals about Coronavirus (COVID-19).

[14] WHO WHO announces COVID-19 outbreak a pandemic.

[15] Almutairi KM, Al Helih EM, Moussa M, Boshaiqah AE, Saleh Alajilan A, Vinluan JM (2015). Awareness, Attitudes, and Practices Related to Coronavirus Pandemic Among Public in Saudi Arabia. Family & Community Health.

